# Multiplexed detection of eight respiratory viruses based on nanozyme colorimetric microfluidic immunoassay

**DOI:** 10.3389/fbioe.2024.1402831

**Published:** 2024-05-16

**Authors:** Feng Wu, Defeng Cai, Xueying Shi, Ping Li, Lan Ma

**Affiliations:** ^1^ Institute of Biopharmaceutical and Health Engineering, Tsinghua Shenzhen International Graduate School, Tsinghua University, Shenzhen, China; ^2^ Shenzhen Institute for Drug Control, Shenzhen, China; ^3^ Department of Clinical Laboratory (Pathology) Centre, South China Hospital of Shenzhen University, Shenzhen, China; ^4^ State Key Laboratory of Chemical Oncogenomics, Tsinghua Shenzhen International Graduate School, Tsinghua University, Shenzhen, China; ^5^ Institute of Biomedical Health Technology and Engineering, Shenzhen Bay Laboratory, Shenzhen, China

**Keywords:** respiratory virus, nanozyme, microfluidics, immunoassay, multiplexed detection

## Abstract

Pandemics caused by respiratory viruses, such as the SARS-CoV-1/2, influenza virus, and respiratory syncytial virus, have resulted in serious consequences to humans and a large number of deaths. The detection of such respiratory viruses in the early stages of infection can help control diseases by preventing the spread of viruses. However, the diversity of respiratory virus species and subtypes, their rapid antigenic mutations, and the limited viral release during the early stages of infection pose challenges to their detection. This work reports a multiplexed microfluidic immunoassay chip for simultaneous detection of eight respiratory viruses with noticeable infection population, namely, influenza A virus, influenza B virus, respiratory syncytial virus, SARS-CoV-2, human bocavirus, human metapneumovirus, adenovirus, and human parainfluenza viruses. The nanomaterial of the nanozyme (Au@Pt nanoparticles) was optimized to improve labeling efficiency and enhance the detection sensitivity significantly. Nanozyme-binding antibodies were used to detect viral proteins with a limit of detection of 0.1 pg/mL with the naked eye and a microplate reader within 40 min. Furthermore, specific antibodies were screened against the conserved proteins of each virus in the immunoassay, and the clinical sample detection showed high specificity without cross reactivity among the eight pathogens. In addition, the microfluidic chip immunoassay showed high accuracy, as compared with the RT-PCR assay for clinical sample detection, with 97.2%/94.3% positive/negative coincidence rates. This proposed approach thus provides a convenient, rapid, and sensitive method for simultaneous detection of eight respiratory viruses, which is meaningful for the early diagnosis of viral infections. Significantly, it can be widely used to detect pathogens and biomarkers by replacing only the antigen-specific antibodies.

## Introduction

Acute respiratory tract diseases (ARDs) are often caused by viral infections and are one of the primary causes of morbidity and mortality related to communicable diseases worldwide (WHO, 2009; Diseases et al., 2020). One of the major features of an ARD is collaborative infection by multiple viruses (Renois et al., 2010; Pigny et al., 2021; Jiang et al., 2022), which poses challenges in diagnosis and treatment. The main respiratory viruses in such cases include the influenza virus, coronavirus, respiratory syncytial virus, human bocavirus, human metapneumovirus, adenovirus, human parainfluenza viruses, and rhinovirus, which impose a huge burden on the health system (Dhanasekaran et al., 2022; Haney et al., 2022). Therefore, it is of great significance to control the transmission of respiratory viruses, especially during the early stages of infection. Furthermore, diagnosis during the early stages of infection can help determine the basis of clinical treatment while reducing the development of severe cases. However, the diversity of respiratory virus species and their subtypes could result in insufficient diagnoses because of their common symptoms. At present, the frequently used detection methods for respiratory viruses include viral nucleic acid detection and viral antigen detection (Ren et al., 2023). Standard quantitative RT-PCR and multiplexed RT-PCR are widely used in diagnostic laboratories to detect viral nucleic acids with high sensitivity (Lee and Suarez, 2004; You et al., 2017; Corman et al., 2020; Goto et al., 2023). Next-generation sequencing (NGS) is a mean of the high-throughput readout (Yelagandula et al., 2021; Gaston David et al., 2022; Ramos et al., 2023), but these methods have limitations such as cumbersome procedures and poor timeliness for detection. Hence, there is urgent need for an ultrasensitive yet rapid detection technology for simultaneous diagnosis of multiple respiratory viruses.

Rapid and simple detection methods, such as the lateral flow immunoassay (LFIA), played a vital role during the COVID-19 pandemic (Castrejón-Jiménez et al., 2022; Filchakova et al., 2022). Noble metal catalytic nanoparticles (NPs) were widely used in the LFIA as they could regulate the local surface plasmon resonance effect though the morphology and deposition of platinum, which presents as dark blue or black, to improve the colorimetric sensitivity. These NPs showed high catalytic efficiency, extraordinary stability in complex environments, and facile production to emerge as promising materials for signal amplification in colorimetric immunoassays (Gao et al., 2017; Loynachan et al., 2017; Wrasman et al., 2018; Lin et al., 2021; Zhu et al., 2024). In this study, bioinformatics analysis and nanomaterial optimization were combined to systematically improve the sensitivity, specificity, and detection rate of viral diagnosis. Furthermore, the labeling of nanozymes was optimized to significantly enhance the sensitivity of detection of respiratory virus antigens. By combining the aforementioned efforts with a microfluidic chip design, rapid and sensitive detection of respiratory virus was achieved along with visual detection of the antigen with a limit of detection (LOD) of 0.1 pg/mL and high detection rate. This study provides a convenient multiplexed microfluidic immunoassay chip based on nanozymes for the detection of eight respiratory viruses, thus laying a foundation for the early diagnosis of other viruses and biomarkers.

## Materials and methods

### Reagents

Disodium hydrogenphosphate (Na_2_HPO_4_, ≥99.0%), sodium phosphate monobasic monohydrate (NaH_2_PO_4_∙H_2_O, 98%–102.0%), sodium carbonate (Na_2_CO_3_, ≥99.0%), sodium bicarbonate (NaHCO_3_, 99.7%–100.3%), 2-(N-morpholino) ethanesulfonic acid (MES), and Tween-20 were purchased from Sangon Biotech (Shanghai, China). Gold (III) chloride tetrahydrate (HAuCL_4_∙4H_2_O, Au>47.8%) was purchased from Beijing Huawei Ruike Chemical (Beijing, China). Hydrogen hexachloroplatinate (IV) hexahydrate (H_2_PtCL_6_∙6H_2_O, 99%) was purchased from Energy Chemical (Shanghai, China). Polyvinylpyrrolidone (PVP, molecular weight: 10kDa) was purchased from Tokyo Chemical Industry. Streptavidin, sulfo-NHS-LC-biotin, 1-ethyl-3-[3-dimethylaminopropyl] carbodiimide hydrochloride (EDC), and N-hydroxysulfosuccinimide (sulfo-NHS) were purchased from Thermo Fisher Scientific (United States). Sodium citrate tribasic dihydrate (99%), L-ascorbic acid (AA, 99%), bovine serum albumin (BSA), and casein block buffer were purchased from Sigma-Aldrich. Polydimethylsiloxane (PDMS; Sylgard 184) was purchased from Dow Corning (United States); photoresist (SU-8 2050) was purchased from MicroChem Corp. (MA, United States); carboxyl-functionalized magnetic beads (MBs) with an average diameter of 10 µm were purchased from Suzhou Nanomicro Technology (China); one-step TMB substrate solution was purchased from Beijing Makewonderbio (Beijing, China). The respiratory virus proteins and antibodies (mAb IgG) were obtained from the Institute of Biopharmaceutical and Health Engineering, Tsinghua University. All aqueous solutions were prepared using deionized (DI) water with a resistivity of 18.2 MΩ·cm.

### Anti-respiratory virus monoclonal antibodies

Monoclonal antibodies with high specificities against influenza A virus, influenza B virus, respiratory syncytial virus (RSV), SARS-CoV-2, human bocavirus (HBoV), human metapneumovirus (HMPV), adenovirus (AdV), and human parainfluenza viruses (HPIVs) were produced and identified, and their target proteins are shown in Table 1. The antibodies against the nucleocapsid proteins of the HPIVs cannot cross-react with other subtypes from among HPIV-1, HPIV-2, and HPIV-3, so specific antibodies were selected for each subtype and mixed to detect these three subtypes.

**TABLE 1:** Monoclonal antibodies against respiratory viruses and their target proteins.

Monoclonal antibody	Respiratory virus	Targeted antigen protein
Anti-IVA-1, 2	Influenza A virus (IAV)	Nucleocapsid protein
Anti-IVB-1, 2	Influenza B virus (IBV)	Nucleocapsid protein
Anti-RSV-1, 2	Respiratory syncytial virus (RSV)	Nucleocapsid protein
Anti-SARS2-1, 2	SARS-CoV-2	Nucleocapsid protein
Anti-HBoV-1, 2	Human bocavirus (HBoV)	Major capsid protein VP1
Anti-HMPV-1, 2	Human metapneumovirus (HMPV)	Nucleocapsid protein
Anti-AdV-1, 2	Adenovirus (AdV)	Hexon protein
Anti-HPIV1-1, 2	Human parainfluenza virus 1 (HPIV1)	Nucleocapsid protein
Anti-HPIV2-1, 2	Human parainfluenza virus 2 (HPIV2)	Nucleocapsid protein
Anti-HPIV3-1, 2	Human parainfluenza virus 3 (HPIV3)	Nucleocapsid protein

### Synthesis of Au@Pt nanozyme

Platinum-decorated Au@Pt nanozyme (Au@Pt NPs) was synthesized as described in a previous study (Wu et al., 2022). In brief, 15-nm gold NPs were used as the seed and mixed with 190 mL of purified water, followed by the addition of 4 mL of PVP (20% w/v). This solution was mixed strongly for 2 min so that the polymer could coat and stabilize the AuNP seeds; then, 8 mL of L-ascorbic acid (10% w/v) was added to the solution and mixed for 1 min. Next, approximately 1600 μL of chloroplatinic acid hydrate (0.5 M) was added to the mixture and mixed for 1 min; this solution was immediately heated to 65°C in an oil bath without stirring for 30 min until its color turned brown/black. The synthesized Au@Pt NPs were cooled to room temperature, purified by centrifugation (15 min, 14,000*g*), and resuspended in distilled water three times. Finally, Au@Pt NPs of size 75 nm were synthesized.

### Preparation of nanozyme–antibody conjugates

In this study, antibodies were allowed to form coordination bonds with sulfur groups that were attached to the surfaces of the Au@Pt NPs. Briefly, 10 mg of DTT was dissolved per milliliter of water, and this solution was added to each labeling antibody at a concentration of 25 μL/mg. The mixtures were then incubated at 4°C for 30 min, following which the excess DTT was removed by ultrafiltration centrifugation using 20 mM sodium phosphate buffer. The modified L-mAbs should be used immediately in conjugation reactions. Next, 100 μL of 75 nm Au@Pt NPs (2.5 nM) was mixed with 400 μL of 20 mM sodium phosphate buffer, followed by addition of 54 μg of modified L-mAbs; this mixture was incubated for 2 h using gentle rotations at room temperature. The modified particles were subsequently blocked using 200 μL of the blocking solution (phosphate buffer containing 1 wt% casein block and 1 wt% glutathione) for 30 min with gentle rotations at room temperature. The excess reagents were removed through three washing steps by centrifugation (15 min, 14,000*g*) using 20 mM sodium phosphate buffer. Finally, the Au@Pt NPs-mAbs were resuspended in a storage buffer at a concentration of 500 p.m.

### Antibody functionalization of MBs

Antibody-functionalized MBs were prepared in accordance with the streptavidin–biotin system, and streptavidin-conjugated MBs were prepared according to the EDC/NHS method. Briefly, 100 mg of carboxyl-functionalized MBs were separated using a magnet for 1 min and washed with MES buffer three times before being activated by 2 mM sulfo-NHS and 5 mM EDC for 30 min at 37°C with gentle rotations. The activated MBs were separated using a magnet, washed with MES buffer three times, and redispersed in 50 mM borate buffer to react with 1.5 mg streptavidin for 3 h at 37°C with gentle rotations. The residual active coupling sites or non-specific binding sites were blocked with a blocking solution for 30 min at 37°C, and the streptavidin-coated MBs were finally washed four times with 20 mM sodium phosphate buffer. Biotinylation of each coated antibody (C-mAb) was performed. Briefly, C-mAbs underwent dialysis against phosphate-buffered saline (PBS) and was diluted to a concentration of 2 mg/mL. Then, sulfo-NHS-LC-biotin was dissolved in DMSO at a concentration of 10 mM, and approximately 13.5 μL of the sulfo-NHS-LC-biotin solution was added to 1 mg of the C-mAbs solution and reacted for 24 h at 4°C. The unreacted biotinylation reagent and byproducts were removed by dialysis against PBS for 24 h at 4°C to purify the biotinylated antibodies. Then, the streptavidin-coated MBs and 0.3 mg of the biotinylated antibodies in PBS were incubated for 1 h at room temperature with gentle rotations. The antibody-coated MBs were separated using a magnet and washed four times using 20 mM sodium phosphate buffer. Finally, the MBs-streptavidin-mAbs were resuspended in phosphate buffer with 1 wt% BSA.

### Preparation of multiplexed microfluidic immunoassay chips

**FIGURE 1:**
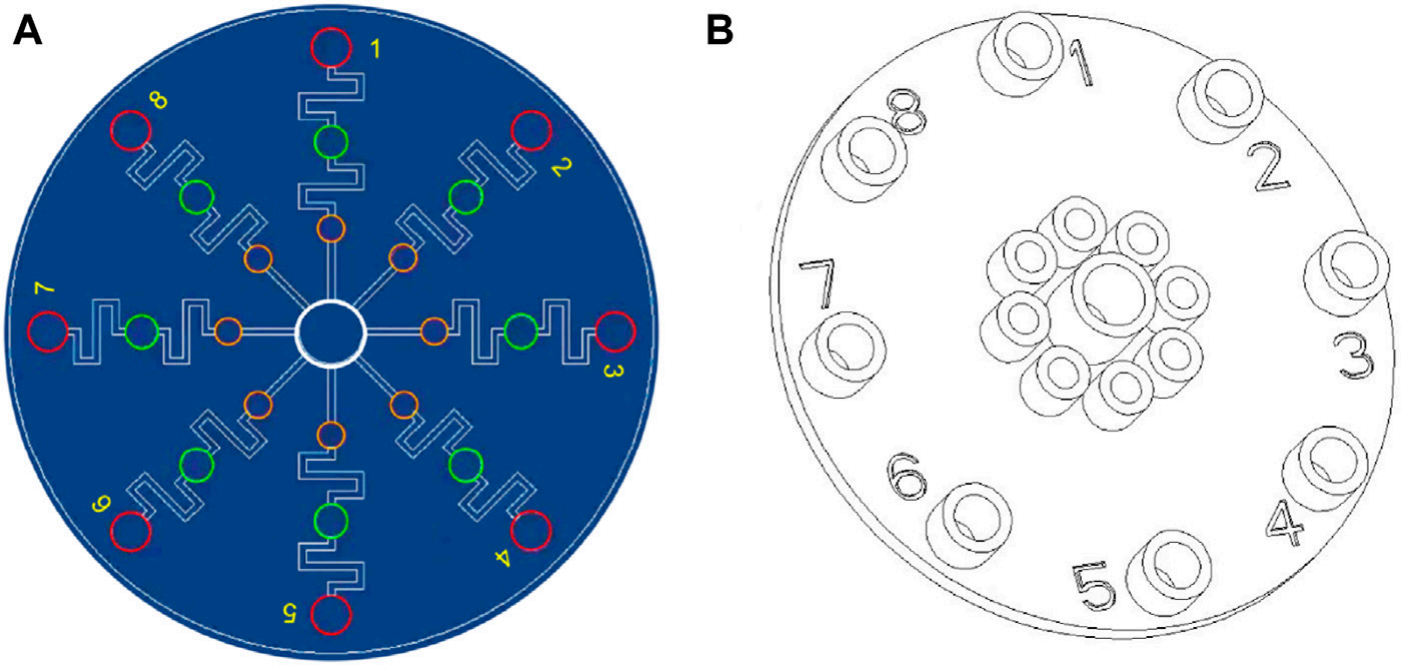
Schematic illustrations of the multiplexed microfluidic immunoassay chip: **(A)** AutoCAD layout of the microchannel structure; **(B)** top structural view of the microfluidic chip.

The microfluidic chips were designed using AutoCAD 2019 and fabricated as described in a previous study (Wu et al., 2022). The structure of the multiplexed microfluidic immunoassay chip is shown in Figure 1A and consists of one sample Luer inlet port at the middle (white loop) surrounded by eight reaction Luer ports (yellow loop) and eight Luer outlet ports (red loop). There is a wash chamber (green loop) in each path between the reaction Luer port and outlet port. The inlet and outlet ports are connected to the chamber by channels of 1000 μm width, and a height of 100 μm was considered for the chamber. The structural overview of the microfluidic chip is given in Figure 1B.

### Analytical procedure

**FIGURE 2:**
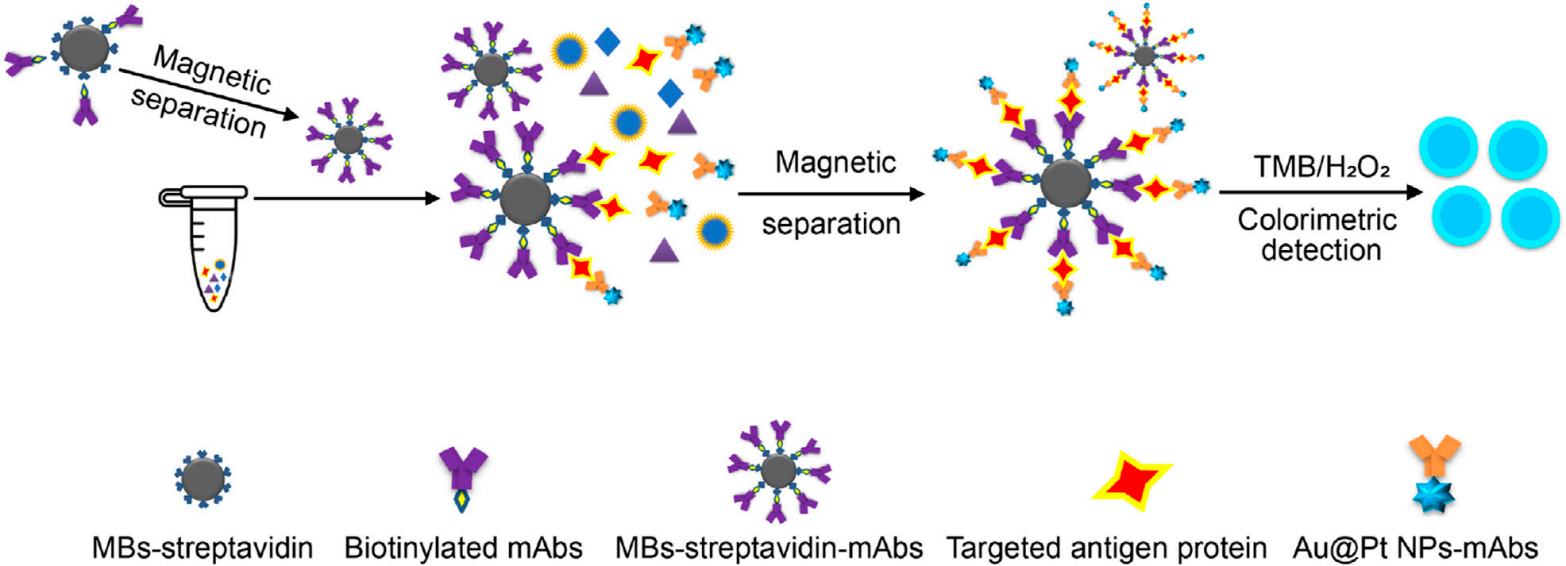
Schematic representation of the colorimetric formation of the sandwich immunoassay.

For the immunoassay preparation, each respiratory-virus-related nanozyme–antibody conjugate (Au@Pt NPs-mAbs) and antibody-functionalized-MBs (MBs-streptavidin-mAbs) were added to the corresponding reaction Luer ports of the microfluidic chip and freeze-dried overnight in vacuum. Upon removal from the freeze-dryer, the chips were stored in a sealed plastic bag filled with silica bead desiccant at room temperature. The two selected antibodies used in the formulation of the nanozyme–antibody conjugate (Au@Pt NPs-mAb) and antibody-functionalized-MBs (MBs-streptavidin-mAb) were designed to specifically recognize the same antigen protein of each virus. Figure 2 shows the formation of the sandwich immunoassay and its colorimetric formation.

The test procedure begins with placing the chip on the magnet, followed by addition of 400 µL of the standard protein solution or sample lysate into the sample Luer inlet port; then, the T-type silicone rubber plug is placed over the inlet and pressed till it touches the bottom. The samples are equally distributed to the eight reaction Luer ports, where the freeze-dried Au@Pt NPs-mAbs and MBs-streptavidin-mAbs recognize and bind with the antigen proteins in the samples to form a sandwich structure. After incubation for 30 min, T-type silicone rubber plugs are placed over each of the reaction Luer ports and pressed to sufficiently push the reaction mixture toward the wash chamber, where the MBs-complex is isolated from the reaction mixture by the magnet. Next, absorbent paper is placed in the eight Luer outlet ports, and approximately 600 µL of the wash buffer is injected from the sample Luer inlet port using a syringe to wash the MBs-complex, followed by removing the magnet and absorbent paper. Finally, 400 µL of the one-step TMB substrate solution is injected from the sample Luer inlet port using a syringe, and the results are detected after incubation for 1–10 min. Usually, the analytical procedure is completed within 40 min. The TMB substrate solution flushes the MBs-complex in the wash chamber into the Luer outlet port to react with the captured Au@Pt NPs and develop color. These developed colors can be conveniently examined and judged visually, or the mixture can be transferred to a microwell plate for absorbance detection at 630 nm using a microplate reader.

## Results

### Properties of antigen-specific monoclonal antibodies

**FIGURE 3:**
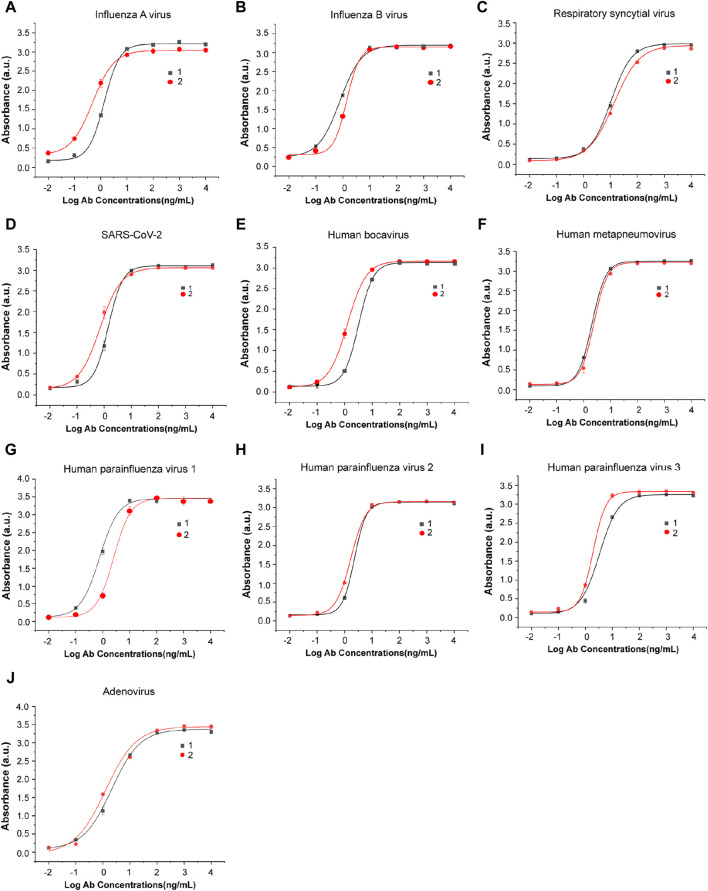
Antibody–antigen binding and titer identification of mAbs against eight types of respiratory viruses. Two specific antibodies were selected and detected by indirect ELISA for each virus: **(A)** influenza A virus, **(B)** influenza B virus, **(C)** respiratory syncytial virus, **(D)** SARS-CoV-2, **(E)** human bocavirus, **(F)** human metapneumovirus, **(G)** human parainfluenza virus 1, **(H)** human parainfluenza virus 2, **(I)** human parainfluenza virus 3, and **(J)** adenovirus. n = 3 biological replicates. The data are mean ± SEM.

The monoclonal antibodies used in this study were screened, expressed, and purified in the lab. Two antigen-specific antibodies with high affinity were selected for each virus. The binding capacities of these antibodies with their antigens were detected by indirect ELISA, and the results are shown in Figure 3. These antibodies were purified in a high titer and can recognize their antigens even at low concentrations, showing their sensitivity and potential for viral antigen detection.

### Properties of Au@Pt NPs

**FIGURE 4:**
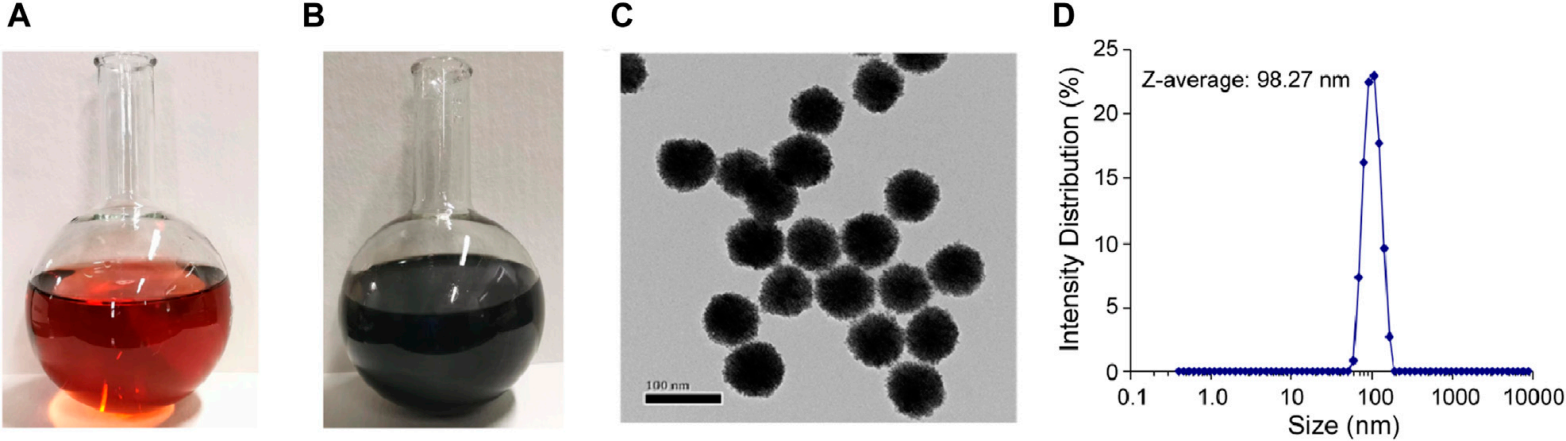
Properties of Au@Pt NPs. Images of Au@Pt NP reaction components **(A)** before and **(B)** after heat treatment. **(C)** TEM images of Au@Pt NPs. Scale bar, 100 nm. **(D)** Nanoparticle size detection by dynamic light scattering (DLS) experiments.

The Au@Pt NPs were prepared with controlled sizes. The color of the platinum-decorated Au NPs changed from red (Figure 4A) to dark brown (Figure 4B) after heat treatment. Then, these Au@Pt NPs were investigated using transmission electron microscopy (TEM) for their structure and size (Figure 4C). The average size of Au@Pt NPs was further detected by dynamic light scattering (DLS) and was found to be approximately 98.27 nm (Figure 4D).

### Properties of multiplexed microfluidic immunoassay chips

**FIGURE 5:**
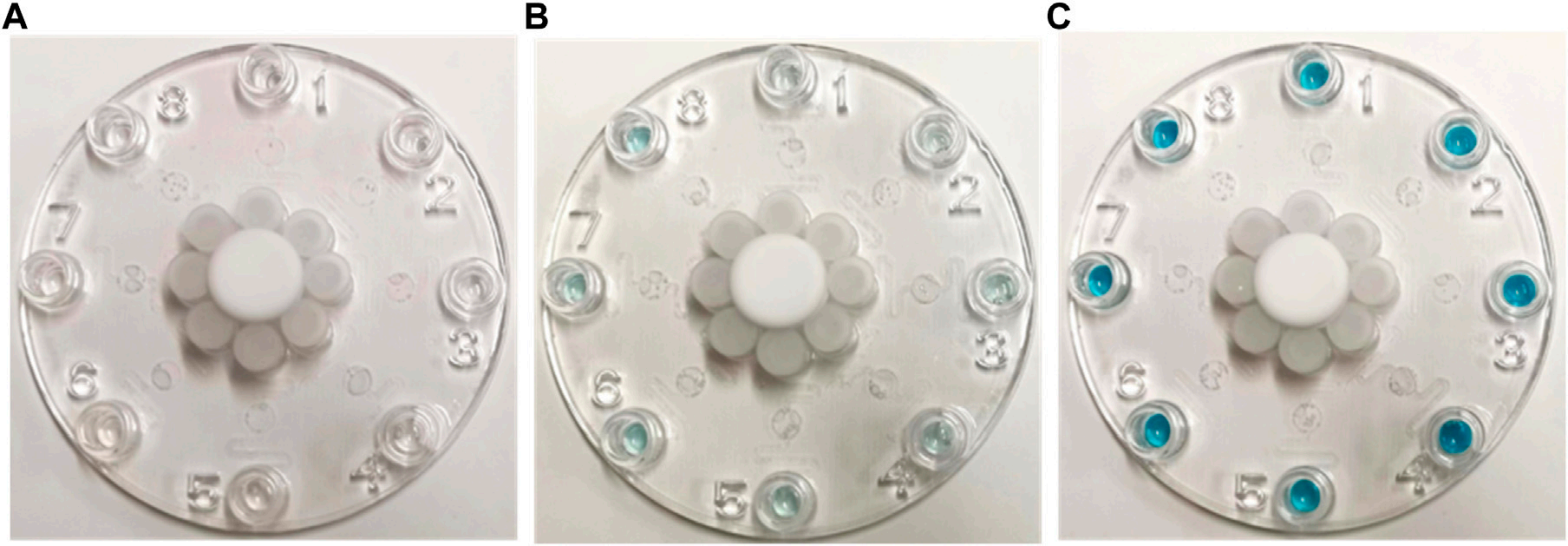
Images of multiplexed microfluidic immunoassay chip detection of different protein concentrations at **(A)** 0, **(B)** 0.1, and **(C)** 100 pg/mL. The positive results are shown in blue at the Luer outlet ports.

**FIGURE 6:**
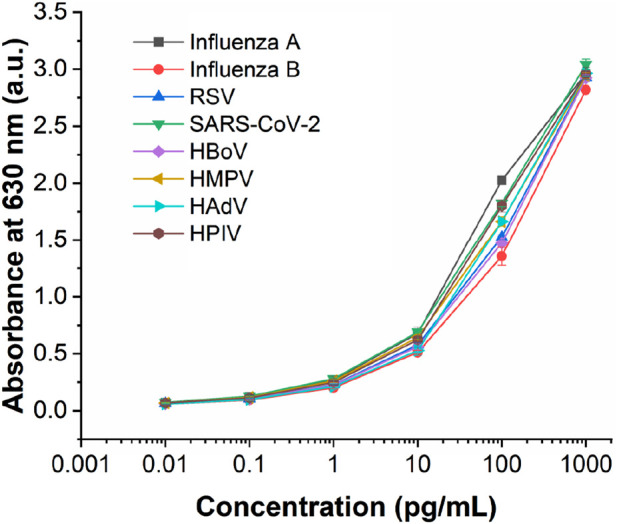
Analytical performance of the multiplexed microfluidic immunoassay chip for detecting the proteins of eight viruses, with absorbance intensities obtained at different protein concentrations ranging from 0.01 to 1000 pg/mL. n = 3 biological replicates. The data are mean ± SEM.

This multiplexed microfluidic immunoassay chip was designed for the simultaneous detection of eight respiratory viruses, namely, influenza A virus, influenza B virus, RSV, SARS-CoV-2, HBoV, HMPV, AdV, and HPIVs. The sensitivity of this chip was determined by testing recombinant protein standard samples at concentrations of 0.01–1000 pg/mL. The results of these standard samples could be inferred visually and also measured using a microplate reader at an absorbance of 630 nm. With the help of the nanozyme, the differential color intensity between the limit concentration and negative control was easily distinguishable via healthy color vision. As shown in Figure 5, the color intensity of the 0.1 pg/mL protein could be distinguished from the negative control visually. For detection with the microplate reader, the test limit concentration of each protein was as low as 0.1 pg/mL (Figure 6). These data indicate that the multiplexed microfluidic immunoassay chip can conveniently detect the eight viruses with high sensitivity.

### Specificity of multiplexed microfluidic immunoassay chip

**FIGURE 7:**
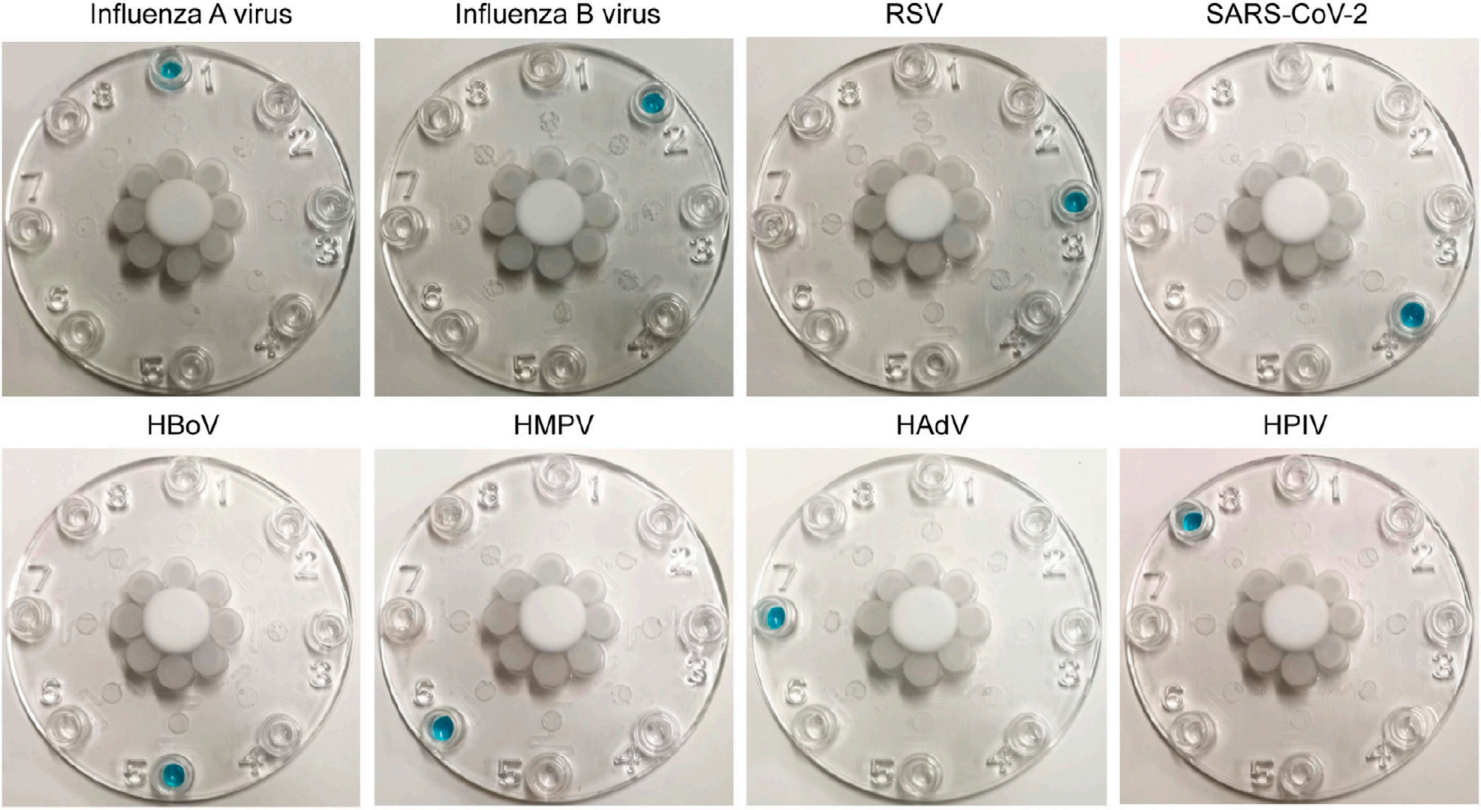
Cross reactions between the eight respiratory pathogens. Approximately 40 pg of protein from each virus in 400 μL of solution (100 pg/mL) was injected into the sample Luer inlet port at the middle, and the results were observed at the Luer outlet ports. The 1–8 Luer outlet ports of each chip show the detection results of influenza A virus (Luer 1), influenza B virus (Luer 2), respiratory syncytial virus (Luer 3), SARS-CoV-2 (Luer 4), human bocavirus (Luer 5), human metapneumovirus (Luer 6), human adenovirus (Luer 7), and human parainfluenza viruses (Luer 8). The positive results are shown in blue.

The specificity and cross reactivity of the multiplexed microfluidic immunoassay chip was analyzed by detecting the conserved nucleocapsid proteins of the influenza A virus (subtype H1N1), influenza B virus (subtype Yamagata strain), RSV (Long strain), SARS-CoV-2 (B.1.1.529 BA.1 and omicron strains), HMPV, HPIVs (subtypes 1, 2, 3, and 4), VP1 protein of HBoV, and HP protein of AdV. These proteins of each virus were mixed for detection; as shown in Figure 7, the multiplexed microfluidic immunoassay chip could detect each virus specifically without cross reactivity with the other seven viruses. Furthermore, all eight respiratory pathogens were detected successfully, demonstrating that the multiplexed microfluidic immunoassay chip could detect these viruses with high sensitivity and specificity.

### Sample test

The studies involving human samples were reviewed and approved by the Bioethics Committee (BEC) of Tsinghua Shenzhen International Graduate School. In this study, human throat swab samples (n = 315) were collected from Shenzhen Children’s Hospital and were diagnosed by real-time RT-PCR/PCR, whose results are shown in Table 2. These samples were inactivated before testing with the multiplexed microfluidic immunoassay chip. Before detection, approximately 300 μL of the human throat swab sample and 300 µL of the lysis buffer (PBS containing 0.5% NP40, 0.2% Tween 20%, and 0.2% LNAC, pH 7.4) were mixed and incubated for 1 min, and the lysate was directly tested using the microfluidic chip immunoassay. As shown in Table 2, the multiplexed microfluidic immunoassay chip could efficiently recognize all eight respiratory viruses in the throat swab samples. Compared with the results of the real-time RT-PCR assay, the microfluidic chip immunoassay had accuracies of 100%, 96.5%, 100%, 100%, 96%, 96%, 96.5%, and 92% for the detection of influenza A virus, influenza B virus, RSV, SARS-CoV-2, HBoV, HMPV, AdV, and HPIVs, respectively. The positive coincidence rate between the multi respiratory virus microfluidic immunoassay and real-time RT-PCR results was 97.2%, negative coincidence rate was 94.3%, and total coincidence rate was 98.1%.

**TABLE 2:** Sample testing results from the multiplexed microfluidic immunoassay chip and real-time RT-PCR/PCR.

Respiratory virus	Number of samples	Microfluidic chip immunoassay (P/N)	Real-time RT-PCR/PCR (P/N)
Influenza A	30	30/0	30/0
Influenza B	30	29/1	30/0
RSV	30	30/0	30/0
SARS-CoV-2	20	20/0	20/0
HBoV	25	24/1	25/0
HMPV	25	24/1	25/0
AdV	30	29/1	30/0
HPIV	25	23/2	25/0
Negative sample	100	0/100	0/100

## Discussion and conclusion

Given the diversity of viruses involved in acute respiratory tract infections and similarities between the clinical symptoms caused by different pathogens, it is difficult and insufficient to make judgments based on clinical manifestations and chest imaging (Lim et al., 2006). However, there are significant differences in the pathological course and treatment for different respiratory viruses. Therefore, it is of great significance to rapidly and accurately diagnose and identify pathogens to help determine their clinical treatments. Thus, numerous studies have been performed to develop multiplexed detection methods for diagnosing pathogens, especially in multiplexed infections cases (Chung et al., 2021; Banerjee et al., 2022). However, it is difficult for these assays based on nuclei acids to detect RNA or DNA viruses simultaneously, such as the DNA viruses like AdV and HBoV along with RNA viruses like the influenza viruses and HPIVs.

In the present study, nanozyme was combined with microfluidic technology to establish a simple and visual rapid detection technology for multiple respiratory viruses. The platinum-decorated Au nanozyme (Au@Pt NPs) that was previously designed and successfully used for SARS-CoV-2 detection was applied to improve the sensitivity of the multiplexed microfluidic immunoassay chip (Wu et al., 2022). The LOD could be decreased significantly to 0.1 pg/mL to enable visual detection, which was convenient for the early diagnosis of infections. This is important because infection by respiratory viruses show very low titer values at the early stages in some varieties, which may result in imprecise diagnoses. Significantly, the multiplexed microfluidic immunoassay chip could simultaneously determine eight pathogens through eight non-interfering microfluidic paths, rendering the diagnosis more accurate. The antibodies used in this study were against conserved proteins of the viruses to ensure detection of all subtypes of each virus and their mutants, such as the influenza viruses A and B, achieving sufficient diagnoses for multiple viruses. This detection was achieved within 40 min, which greatly improved the effectiveness and provided a new scheme for rapid and sensitive detection of respiratory viruses.

In summary, a multiplexed microfluidic rapid detection method based on nanozymes is proposed for diagnosing multiple respiratory viruses, and this method can be widely used to detect pathogens and biomarkers by replicating antigen-specific antibodies, which provide technical support for the development of point-of-care testing.

## Data Availability

The original contributions presented in the study are included in the article/supplementary material; further inquiries can be directed to the corresponding authors.
